# Pulmonary Edema and Diastolic Heart Failure in the Perioperative Period

**DOI:** 10.1155/2018/5101534

**Published:** 2018-01-23

**Authors:** Galen Royce-Nagel, Kunal Karamchandani

**Affiliations:** ^1^Department of Anesthesia, Critical Care and Pain Medicine, Massachusetts General Hospital, Boston, MA, USA; ^2^Department of Anesthesiology and Perioperative Medicine, Penn State Health Milton S. Hershey Medical Center, Hershey, PA, USA

## Abstract

Heart failure with preserved ejection fraction (HFPEF) is a diagnosis encountered with increasing frequency in the aging population. We present a case of postoperative pulmonary edema in 63-year-old male with HFPEF. This patient highlights the gap in risk stratification with respect to diastolic heart failure.

## 1. Introduction

Heart failure with preserved ejection fraction (HFPEF), or diastolic heart failure (HF), refers to the clinical syndrome of HF coupled with evidence of diastolic dysfunction and is associated with significant mortality and morbidity [1]. The incidence of HFPEF has been variedly described between 30 and 50% among patients with heart failure [2]. The goals of care for perioperative management of these patients include maintenance of adequate preload, slower heart rate to accommodate for adequate diastolic filling time, and avoidance of hypertension to decrease the afterload to the left ventricle. The association of acute myocardial infarction with perioperative HFPEF is rare but can have disastrous consequences. We describe the case of acute HFPEF presenting as a harbinger of myocardial infarction in the perioperative period.

## 2. Case Description

A 63-year-old male, weighing 82 kg (BMI 28), was scheduled for abdominoperineal resection (APR) and right partial hepatectomy for metastatic colon cancer. His past medical history was significant for hypertension, recently diagnosed non-insulin dependent diabetes, a cerebral vascular accident 9 years prior to surgery, an 80-pack year smoking history (quit 20 years prior to surgery), and an episode of acute congestive heart failure with preserved ejection fraction approximately one year before surgery.

At preoperative evaluation, the patient described his functional capacity as excellent (able to climb 2 flights of stairs multiple times a day with no symptoms; worked more than 60 hrs. a week as a mechanic) and denied any symptoms of HF. The physical examination did not reveal any signs of congestive heart failure. Transthoracic echocardiogram (TTE) showed normal left ventricular (LV) size and function (EF of 55%) and no significant valvular pathology. Since the patient was scheduled for an intermediate risk surgery, had no active cardiac conditions, and had good functional capacity, a decision was made to proceed with surgery without further work-up based on guidelines established by the American College of Cardiology and the American Heart association (ACC/AHA).

General anesthesia was induced with propofol and maintained with isoflurane plus an air-oxygen mixture of equal parts. Standard ASA monitors were applied along with invasive hemodynamic monitoring using a right radial arterial line. Patient had a preexisting tunneled central venous catheter that was accessed and 2 large bore IVs were inserted for intravenous access. He received muscle relaxation with intermittent doses of intravenous (i.v.) rocuronium titrated to keep the train of four to less than 2 twitches. He received a total of 2 mg i.v. hydromorphone in divided doses and 1000 mg of i.v. acetaminophen for analgesia.

The surgery lasted approximately eight hours. His estimated blood loss was 400 ml and urine output was 300 ml. He received 7 liters of crystalloid and 1 liter of albumin. His intraoperative fluid management was guided by measurement of stroke volume variation (SVV) via Vigileo™, serum lactate levels, and urine output. He received fluid boluses to keep the SVV less than 13, lactate levels less than 2 mg dl^−1^, and urine output greater than 0.5 ml kg^−1^ per hour. He remained hemodynamically stable throughout the surgery. He did not have any significant electrolyte abnormalities as determined by hourly arterial blood gases (ABGs) during the surgery. The patient's trachea was extubated at the end of the procedure after ensuring adequate reversal of neuromuscular blockade and responsiveness to commands.

Immediately after extubation, he developed hypertension and tachycardia with a high blood pressure of 273/100 mm Hg and heart rate ranging between 120 and 130 beats/minute. His oxygen saturation declined to a low of 89% despite supplemental oxygen and airway recruitment maneuvers. He progressively became agitated and less responsive. Pink frothy sputum was noted upon suctioning of his oropharynx. The patient's trachea was promptly reintubated, and 20 mg of i.v. furosemide was given. He was then transferred to the surgical intensive care unit for further management.

The immediate postoperative chest X-ray revealed bilateral vascular congestion. The electrocardiograph (ECG) showed nonspecific inferior lead changes without ST elevations. The serial troponin I levels were obtained and peaked at 0.5 ng ml^−1^ (normal < 0.12). He received an additional dose of i.v. furosemide and over the next 12 hours his respiratory status improved. After meeting all the necessary criteria, his trachea was extubated. His home doses of aspirin, atorvastatin, and metoprolol were restarted. A repeat TTE done on postoperative day 1 (POD 1) showed an ejection fraction of 60% without any wall motion abnormalities. Despite this, he had another episode of acute pulmonary edema on POD 1 followed by atrial fibrillation (AF) with rapid ventricular response (RVR). He was successfully treated with noninvasive continuous positive airway pressure (CPAP) of 5 cm H_2_O and a dose i.v. furosemide for acute pulmonary edema. For his AF with RVR, he received intermittent doses of i.v. metoprolol to achieve rate control with a target heart rate of <100 beats/minute. His dose of oral metoprolol was also increased from 25 mg every 12 hours to 50 mg every 12 hours.

On POD 4, he developed a third episode of respiratory distress with minimal response to diuretics and antihypertensives. His ECG now showed ST elevations in anterior and inferior leads and his troponin I level was 0.74. He underwent emergent cardiac catheterization with placement of two bare metal stents in the mid-left anterior descending and left circumflex vessels for 80% and 85% occlusion, respectively. The remainder of his hospital course was uneventful and he was discharged home on POD 12.

## 3. Discussion

Our patient with postoperative pulmonary edema due to HFPEF serves to highlight a gap in preoperative risk stratification. He attended our preoperative clinic but due to his excellent functional status and resolved HF did not meet criteria for further work-up. The combination of significant fluid shifts and the hyperadrenergic state associated with perioperative stress unmasked his true cardiac deficit with resultant episodes of pulmonary edema. In reflection, is there anything more that could have been done to recognize his increased risk for cardiac complications?

HFPEF is a well-described entity with prevalence ranging from 1.1 to 1.5% [3]. Studies have reported mixed results for perioperative risk in patients with HF and preserved LVEF [4]. In a recent meta-analysis, patients with HFPEF had a lower all-cause mortality rate than did those with HF and reduced LVEF [5]. However, the absolute mortality rate was still high in patients with HF and preserved LVEF as compared with patients without HF. There is limited data on perioperative risk stratification related to diastolic dysfunction and perioperative HFPEF has traditionally been underestimated and not well described in literature.

The American College of Cardiology and the American Heart association (ACC/AHA) as well as the European Society of Cardiology and European Society of Anesthesiology (ESC/ESA) guidelines recommend the use of risk indices for preoperative cardiac evaluation for noncardiac surgery [4, 6]. The commonly used indices, Goldman, Detsky, and Revised Cardiac Risk Index (RCRI), all include HF as an independent prognostic variable [7–9]. Although a history of HF is considered a significant risk for perioperative complications, there is no recommendation for further cardiac testing in patients who are asymptomatic with good functional status [4]. When patients with a prior episode of decompensated HF who improve both symptomatically and functionally on medical therapy present for elective surgery several months later, as our patient did, the perioperative risk calculated comes out markedly low and does not warrant further testing.

Although a goal directed fluid management strategy was chosen for our patient, we believe that the fluid administration was slightly excessive and in hindsight should have been curtailed. The significant fluid shifts associated with such a major surgical procedure along with the mild tachycardia and hypertension experienced during emergence from anesthesia may have contributed to his initial episode of acute pulmonary edema. Conditions unique to the immediate postoperative period such as pain-induced sympathetic activation, shivering, anemia, hypovolemia, and hypoxia may alter the myocardial oxygen balance [10] and further predispose patients with diastolic dysfunction to an episode of acute diastolic HF.

The cardiac catheterization findings in our patient were quite significant despite a seemingly normal TTE. Coronary artery disease is present in 40–55% of patients with diastolic HF [11]. The first abnormality induced by epicardial ischemia is reduced ventricular compliance, not wall motion abnormality, ECG changes, or chest pain [12]. Therefore, patients presenting with acute diastolic HF without clinical or ECG evidence of myocardial ischemia may still have significant angiographic ischemic heart disease [13]. Delay to diagnosis of coronary artery disease for our patient was likely due to his normal preoperative exercise tolerance, a clinical picture of postoperative fluid overload, the mild increase in troponin I levels, and a normal postoperative ECG and TTE.

HFPEF is a category of heart failure that is poorly defined and therefore poorly understood. Development of validated preoperative risk indices that focus on this entity would be valuable in the management of our increasingly at risk population. Our patient's clinical course highlights the importance of keeping diastolic dysfunction high on the differential despite preoperative presentation. It also stresses the possibility of perioperative HFPEF presenting as a harbinger of acute myocardial infarction.
